# The creation of the Fundação Brasileira para a Conservação da Natureza

**DOI:** 10.1590/S0104-59702024000100019en

**Published:** 2024-05-17

**Authors:** Juliana da Costa Gomes de Souza, José Luiz de Andrade Franco, José Augusto Drummond

**Affiliations:** i Doctoral candidate, Center for Sustainable Development/Universidade de Brasília. Brasília – DF – Brazil jcgdesouza@gmail.com; ii Professor, Center for Sustainable Development, Department of History/Universidade de Brasília. Brasília – DF – Brazil jldafranco@gmail.com; iii Professor (retired), Center for Sustainable Development/Universidade de Brasília. Brasília – DF – Brazil jaldrummond@uol.com.br

**Keywords:** Environmental history, International Union for Conservation of Nature, Fundação Brasileira para a Conservação da Natureza, Jerônimo Coimbra Bueno (1910-1996, Victor Abdennur Farah (1915-1967, História ambiental, União Internacional para Conservação da Natureza, Fundação Brasileira para a Conservação da Natureza, Jerônimo Coimbra Bueno (1910-1996, Victor Abdennur Farah (1915-1967

## Abstract

This study within the field of environmental history explores the scenario amid which the Fundação Brasileira para a Conservação da Natureza (Brazilian Foundation for Nature Conservation) was founded between 1958 and 1966; this important Brazilian non-governmental organization headquartered in Rio de Janeiro worked at the local, national, and international levels. Primary documentary sources were utilized, along with research of the related literature. The conclusions demonstrate the importance of non-governmental organizations predating this foundation, and the influence of conservationists on its establishment and current work.

## Resumo

Este estudo se insere no campo da história ambiental e tem o objetivo de compreender o cenário de criação, entre 1958 e 1966, da Fundação Brasileira para a Conservação da Natureza, importante organização não governamental ambientalista brasileira, com sede no Rio de Janeiro e atuação local, nacional e internacional. Para isso, utiliza fontes documentais primárias e pesquisa bibliográfica relacionada. As conclusões demonstraram a importância da existência de organizações não governamentais mais antigas que a fundação e a influência de conservacionistas sobre sua criação e sua atuação inicial.

This article in the area of environmental history focuses on the creation of the Brazilian Foundation for Nature Conservation (Fundação Brasileira para a Conservação da Natureza, FBCN). This organization with headquarters in Rio de Janeiro had local, national, and international influence, primarily from the 1960s to the 1980s, on topics related to nature conservation. Here we explore the context in which the FBCN was created, and also examine the relevance of some of its founding members, particularly Jerônimo Coimbra Bueno (1910-1996) and Victor Abdennur Farah, and their relations with the spheres of politics and Brazilian nature conservation between 1958 and 1966. Coimbra Bueno and Farah were conservationists responsible for encouraging the creation of 11 Brazilian national parks during the 1960s, the most important factor in FBCN’s emergence. This period from 1958 to 1966 marks the early stages of the foundation, which was followed by more structured activity, well-defined members, and publication of informative annual reports about nature conservation.

During the 1950s and 1960s, the world had passed through the 1929 financial crisis and the devastation of two World Wars and was now in the midst of the Cold War. From 1945, global consumption of fossil fuels and other raw materials expanded significantly to support accelerated economic and social growth as plans to invest in infrastructure and development projects emerged around the world. In Brazil, this phenomenon was typified in 1956 by the “Plano de Metas,” a goal-driven plan proposed by the government of president Juscelino Kubitschek (1902-1976) which was intended to expand Brazilian GDP by the equivalent of fifty years in only five, alarming a group of citizens concerned about what this might mean in terms of environmental destruction (McNeill, Engelke, 2016; Urban, 2011, originally published in 1998; Worster, 2016).

This group, which later called themselves “conservationists,” created the FBCN in 1958. Although this was done within a strongly developmentalist context, some factors made them relatively successful in their efforts. We must remember that the circumstances were favorable in the country: there were other associations and institutions for nature protection. This made it easier for the members of the FBCN to come together and organize themselves, in administrative terms as well as in terms of the scientific knowledge acquired in exchanges with other organizations and other countries. Furthermore, many members held posts in public administration or politics, which facilitated approaches and dialog with those responsible for decision making. Some results from the influence of FBCN members in the political sphere materialized during the presidencies of Kubitschek and Jânio Quadros (1917-1992) (McCormick, 1992; Urban, 2001, 2011).

To better understand the era when the FBCN was created, its main activities, the influences that shaped it, and to learn more about its founders, we used primary sources including articles from the *Correio da Manhã*, a significant daily newspaper in Rio de Janeiro, and the statutes of the organization, which were written in 1960 and published in the *Diário do Congresso Nacional* and address the foundation’s organization, objectives, and symbol. Much of the information in the statutes was repeated in the 1966 edition of the *Boletim da FBCN*, the first of 24 annual volumes published by the FBCN until 1989. These reports addressed nature conservation and related practices in Brazil and around the world. Urban (2011) also gathered material that can be used as a primary source in her transcriptions of interviews with some prominent FBCN members.

Additionally, we consulted secondary sources to expand the context of our research. McNeill and Engelke (2016) conducted a global exploration of the period starting in 1945, when human impacts on nature intensified through accelerated economic growth and more significant consumption of natural resources. Sena (2018) deepened discussions on the creation of the International Union for the Protection of Nature (IUPN) and its subsequent renaming as the International Union for Conservation of Nature (IUCN). Within the national sphere, Dean (1996) and Urban (2001, 2011) addressed the environmental destruction suffered by Brazil since the earliest human occupations and how this led to plans for nature protection and conservation over the long term. Franco and Drummond (2009a) examined the relationship between protecting nature and constructing national identity from the 1920s to 1940s in Brazil. Duarte (2010) described natural protection during this same period, which had a direct impact on the generation of conservationists which comprised the FBCN. Hochstetler and Keck (2007) and Pádua (2012, 2018) offered a panoramic view of the history of environmentalism in Brazil, especially during the “great acceleration” in the period following Second World War identified by McNeill and Engelke (2016).

Other texts focused more directly on the work of the FBCN and/or its members. The groundbreaking master’s thesis by Borges (1995) described its organization’s structure and included interviews with important members such as Harold Edgard Strang. Franco and Drummond (2009b, 2013) directly addressed the role played by the FBCN in nature conservation from 1958 to 1992. Gonçalves (2021) and Maia and Franco (2021) studied the role of the agronomist Augusto Ruschi (1915-1986), a researcher at the National Museum of Rio de Janeiro (MNRJ) and founder and director of the Professor Mello Leitão Museum of Biology (MBML), in the history of conservation. Lopes and Franco (2020) investigated the creation of Araguaia National Park and emphasized the importance of senator Jerônimo Coimbra Bueno in bringing it into being; Ribeiro (2020) also demonstrated Coimbra Bueno’s relevance in the creation of Chapada dos Veadeiros National Park. Silva (2017) described the emergence of the Federal Forest Council (Conselho Florestal Federal, CFF) and its presidencies, especially that of the agronomist Victor Abdennur Farah, the first executive director of the FBCN. Finally, Prado (2011) and Pereira (2013) investigated Henrique Luís Roessler (1896-1963), who was part of a movement in the state of Rio Grande do Sul to protect nature which established connections with the FBCN. The authors cited here are among those who worked most directly on topics related to the FBCN, its history, its activities, or those of its members, although they did not specifically focus on its creation or even its foundation per se. The FBCN generally appears in these works as a contributor to nature conservation. Facts on where and when it was created and its main objectives are repeated, with little additional information. This study intends to fill gaps related to the period predating the establishment of the FBCN, as well as its creation itself.

We also point out some incoherencies that have been published about the FBCN. Hochstetler and Keck (2007) reported that the organization was founded by 12 members, while the *Correio da Manhã* and the *Boletim da FBCN* from 1966 show that there were 14 founders. Meanwhile, Diegues (2008) classifies the FBCN as preservationist, ignoring the fact that at its founding the institution mixed conservationism and preservationism. In fact, this conflict between preservationism and conservationism was not seen in Brazil; in the United States, a more romantic and stricter protection of nature involving the maintenance of areas without the presence of humans and an intrinsic transcendental value attributed to nature became known as preservationism, and found its main proponent in the Scottish-born American writer John Muir (1838-1914). Muir contrasted with conservationism, advocated by the American forester Gifford Pinchot (1865-1946), who supported wise use of natural resources and productive activities in protected areas.

To better understand this debate between conservation and preservation, Maia and Franco (2021) returned to the influence of the naturalist Alexander von Humboldt (1769-1859) in redefining nature in western thought, which in turn molded ecological thinking and biogeography. Humboldt recovered romantic contemplation of the wilderness and influenced a variety of thinkers such as Henry David Thoreau (1817-1862), John Muir, and even Gifford Pinchot through a more holistic understanding of nature. In this way, despite the schism between Muir and Pinchot, they were both affected by the romantic influence, as was the American forester Aldo Leopold (1887-1948). Leopold’s understanding of conservation work shifted from managing the wilderness from the time that he broadened his understanding of ecology and began to “think like a mountain,” or understand the whole as Humboldt did. While Thoreau, Muir, and Leopold can be seen as linked to a conservationism with preservationist leanings (or simply preservationism) that attributes an intrinsic biocentric or even ecocentric value to species, Pinchot was a conservationist and more concerned with the wise use of natural resources, classified as an anthropocentric bias. These characteristics were mixed within the FBCN, and do not permit us to classify the organization as either preservationist or conservationist.

This article is divided into three sections, as well as this introduction and a conclusion. In the first section we analyze initial efforts to protect and conserve nature in Brazil. The second focuses on the creation of the FBCN, its founders, and the processes and agreements that were necessary to bring it into being. In the third section we concentrate on the work of the FBCN’s conservationists, more specifically Jerônimo Coimbra Bueno and Victor Abdennur Farah. In the conclusion, we highlight the importance of non-governmental organizations that existed prior to the FBCN and the role played by their members who were able to influence decisions in the political sphere.

## Brazil’s first societies and institutions for protecting and conserving nature

This section describes some events in the 1960s and previous decades. We start with the environmental degradation that accelerated in international as well as domestic contexts from 1945 onward. Discussions related to the destruction of nature led to measures, legislation, and the creation of institutions to protect nature, as well as activism by people linked to this cause. Here we have sought to clarify this context in order to highlight the fact that the creation of the FBCN was facilitated by the prior existence of nature protection and conservation activities.

The global scenario from the FBCN emerged is located within a period of intensified use of fossil fuels and other natural resources as well as exponential population growth known as the “great acceleration.” This phase began between 1945 and 1950, after two World Wars and a long economic recession stemming from the 1929 collapse and proceeding at an almost unsustainable pace until the present day. The idea of conservation was associated with development, permitting a correlation between the creation of national parks, economic development, and national identity. This was an important aspect of the start of the great acceleration that helped conservationists convince politicians and government institutions to create protected areas in Brazil. Another important issue is the Cold War (1947-1991), a period of diplomatic and geopolitical tensions between two large blocs which drove a race for development and economic growth synonymous with modernity and civilization, to the detriment of the environment (De Bont, Schleper, Schouwenburg, 2017; Franco, Drummond, 2009b; Mcneill, Engelke, 2016; Worster, 2016).

The resulting environmental devastation concerned those who sympathized with protecting nature globally, and as a result modern environmentalism emerged in contrast with developmentalism. At the start of the twentieth century attempts were made to create an international body for natural protection, but this only occurred in 1948 with the establishment of the IUPN in Switzerland, a hybrid between governmental and non-governmental agencies. Later, in 1956, it became the IUCN. The World Wide Fund for Nature, a non-governmental organization, emerged in 1961 to fund the IUCN, and later funded the FBCN. After its establishment and some assemblies, the members of the IUCN began to seek more precise definitions about national parks and other types of protected areas and held international conferences every ten years (McNeill, Engelke, 2016; McCormick, 1992; Sena, 2018; Urban, 2011).

Without a strict order of creating societies, institutions, and environmental legislation, this type of movement centering around environmental conservation appeared in various countries including Brazil which were influenced by strong discourses on economic development and modernization. Concerns with environmental degradation in Brazil appeared from the late eighteenth century, alongside problems resulting from deforestation and deterioration of the soil. In the nineteenth century, environmental damage was seen in Brazil as the price of delays from the social practices and rudimentary technologies that were the result of colonialism. Various scholars including the naturalist José Bonifácio de Andrada e Silva (1763-1838), a friend and correspondent of Alexander von Humboldt, discussed topics such as the extinction of species in the Amazon, drought in the Northeast of the country, and the risks of monocrop farming (Franco, Drummond, 2009a, 2012; Maia, Franco, 2021; Medeiros, 2006; Pádua, 2012, 2018; Worster, 2016).

This environmental concern became more evident during the 1920s, 1930s, and 1940s, when a group of intellectuals were able to gain notable access to political decision making and the capacity to influence the administration of then-president Getúlio Vargas. This group connected nature protection with notions of constructing Brazilian national identity. One consequence of these efforts was the publishing of a series of environmental decrees in 1934 (the Hunting and Fishing, Forest, and Water Codes) and measures to protect fauna. Shortly afterward, the first national parks in Brazil were created: Itatiaia (1937), Serra dos Órgãos (1939), and Iguaçu (1939). The 1934 Constitution held the central and state governments responsible for protecting “natural beauty” and “monuments of historical or artistic value.” In the 1930s various associations and institutions for nature preservation appeared inside and outside of the government sphere (Franco, Drummond, 2009a, 2012; Medeiros, 2006; Pádua, 2018).

One of the first public government institutions in Brazil directed at natural protection was created in 1921 and regulated in 1925: the Federal Forest Service, part of the Ministry of Agriculture. This institution only began to undertake significant activities in the 1930s with the creation of the first national parks. The following year, outside the sphere of the government, the Society of Friends of the Trees (Sociedade dos Amigos das Árvores) was born at the initiative of Alberto José Sampaio (1881-1946) and Leôncio Correia (1865-1950). Correia founded the *Correio da Manhã* newspaper, where he and others played an important role in disseminating ideas about protecting nature. This society brought together intellectuals, journalists, and politicians who were concerned with the accelerated destruction of nature, and held the First Brazilian Conference on Natural Protection, in 1934, in Rio de Janeiro. This event reflected the type of thinking that became characteristic of civil society organizations and public institutions that focused on protecting nature in Brazil. The topics on the agenda were defense of flora and fauna and natural monuments, and protection and better use of the country’s natural resources. This conference paved the way for the drafting of the Forest Code in 1934 as well as the creation of Brazil’s first national parks (Franco, Drummond, 2009a; Medeiros, 2006; Pádua, 2012; Silva, 2017; Urban, 2001, 2011).

Also within the government sphere, in 1932 the Society of Friends of Alberto Torres was established in Rio de Janeiro, which was intended to contribute to legislative discussions taking place at the National Constituent Congress of 1934. Its founders included the botanist Alberto José Sampaio, director of the MNRJ, and the sculptor, draftsman, professor, and writer Armando Magalhães Corrêa (1889-1944). This society disseminated the ideas of Alberto Torres (1865-1917), a lawyer, politician, and writer who was active in the state of Rio de Janeiro. Torres strongly criticized the destruction of natural resources solely in pursuit of territorial and economic expansions; he proposed a constitutional amendment including an article defending the soil and natural wealth of the country and distribution of land to small-scale owners, and suggested these activities be carried out by a strongly interventionist government. The society was active until 1945 (Franco, Drummond, 2009a; Urban, 2001, 2011; Worster, 2016).

Prior to these societies, there were other civic groups organized around the question of natural protection. Their work was more specific, as a broader environmental critique emerged particularly in the 1950s. But notable among these organizations were the Brazilian Excursionist Center (Centro Excursionista Brasileiro), created in 1919, in Rio de Janeiro, whose guides were licensed as forest guards, the Brazilian Federation for Feminine Progress (Federação Brasileira para o Progresso Feminino), established in 1922 and which counted the biologist Bertha Lutz (1894-1976) among its leaderships, and the Society of Friends of the National Museum, made up of MNRJ staff and established in 1937. It is also important to note that women were pioneers in decrying the use of plumage from birds to decorate clothing and hats manufactured in England. Important figures in Brazil were Magda Renner (1926-2016), who lived in the South of the country and joined the environmentalist cause after attending a lecture by the agronomist José Lutzenberger (1926-2002), who wrote articles for the FBCN bulletins from the 1970s (Dean, 1996; Franco, Drummond, 2009a, 2012; McCormick, 1992; Urban, 2001, 2011).

In 1939, the botanist Frederico Carlos Hoehne (1882-1959) founded the Society of Friends of Brazilian Flora (Sociedade de Amigos da Flora Brasílica), based in São Paulo. The society worked together with the state’s Institute of Botany to promote publications, organize lectures, and influence public opinion in favor of reforestation and biological preserves (Dean, 1996; Franco, Drummond, 2009a, 2012; Urban, 2001, 2011). In the late 1930s and early 1940s, these initiatives, despite their local nature, nevertheless provided experience and organization capacity to the intellectuals concerned with protecting nature.

Brazilian environmentalism was born from the union of external influences combined with domestic knowledge, with strong western characteristics working together with local activities, as mentioned above. Even though developing countries attained significant participation in international conferences on nature conservation from the 1970s, within the Americas the Convention on Nature Protection and Wildlife Preservation in the Western Hemisphere was signed in Washington in 1940. This agreement included the governments of Bolivia, Cuba, El Salvador, Nicaragua, Peru, the Dominican Republic, the United States, Venezuela, and Ecuador as participants in the Pan-American Union; it took effect in 1942 and was approved by Brazil in 1948. Topics discussed included defining national parks, natural monuments, and preserved virgin landscapes (biological preserves) (Carvalho, 1969; De Bont, Schleper, Schouwenburg, 2017; Pádua, 2012, 2018).

In the 1950s and 1960s, national-developmentalist political ideology was widespread in Brazil. President Kubitschek presented his plan of goals, with a focus on occupation of the Central Plateau that updated Getúlio Vargas’s “March to the West” during the New State period meant to promote economic integration and encourage the settlement of vast areas in Brazil’s Midwest and North regions. This plan had strong impacts on nature, since it stimulated accelerated economic development and more widespread occupation of this territory (Fausto, 2013; Franco, Schittini, Braz, 2015; Risério, 2013).

Also during the 1950s, other organizations dedicated to natural protection emerged: in late 1953, the agronomist Augusto Ruschi, who was concerned with the destruction of the Atlantic Forest in Espírito Santo and plans for reforestation with eucalyptus, created the Brazilian Society for Natural Protection (Sociedade Brasileira de Proteção à Natureza, SBPN). Ruschi adhered to the conservationist ideas of previous generations of intellectuals and disseminated them with his publications in the bulletins of the MBML. He can be considered a pioneering communicator of topics that led to intense debates in Brazil and abroad, particularly after the Stockholm Conference in 1972 (Franco, Drummond, 2009a, 2009b, 2013; Gonçalves, 2021; Maia, Franco, 2021; Urban, 2001, 2011).

Another important entity that was active in São Paulo was the Association for the Defense of Flora and Fauna (Associação de Defesa da Flora e da Fauna), which supported the establishment of a public forest preserve in Pontal do Paranapanema, in the far west of the state. This association was created in 1956 by José Carlos Reis de Magalhães (1921-2002), Lauro Pereira Travassos Filho (1918-1989), and Paulo Nogueira Neto (1922-2019); Nogueira Neto became a member of the FBCN in 1966. Later, the name of the association changed to the São Paulo Environmental Defense Association (Associação de Defesa do Meio Ambiente de São Paulo); the name was changed because the association initially had a limited concept of conservation, and its members came to understand that controlling pollution and water and air quality were also part of solutions for environmental issues (Urban, 2001, 2011).

The environmental movement also created societies in southern Brazil with major domestic and even international presence. The Jesuit priest Balduíno Rambo (1906-1961) studied Brazil’s natural parks, decried deforestation to obtain firewood, and criticized rice monoculture in the riparian woodlands in the Taquari-Antas and Caí River basins in the state of Rio Grande do Sul. He suggested creating a forest park in the Upper Uruguai River, and Aparados da Serra, both also in Rio Grande do Sul. But it was Henrique Luís Roessler (1896-1963) of this same state who was able to articulate the movement in this region. Roessler dedicated himself to the environmental movement in Rio Grande do Sul by creating the Union for Nature Preservation (União de Proteção à Natureza) in 1955. He also published chronicles about nature conservation in the *Correio do Povo* newspaper (Prado, 2011; Pereira, 2013; Gritti, 2017; Laroque, 2017; Urban, 2001, 2011).

By the late 1950s, the Brazilian environmental movement had regional activities and leadership. Some of these leaders later met again in natural conservation work at FBCN, such as Lutzenberger and Nogueira Neto, bringing their knowledge and prior experience to activities at the domestic and international scale. Not only were they up to date on Brazilian and international conferences to exchange knowledge on conservation of nature and natural resources (like Harold Edgard Strang, who participated in the sixth Assembly of the IUCN in Athens in 1958, or future FBCN president José Cândido de Melo Carvalho [1914-1994], who wrote on natural conservation in Brazil and abroad in 1969 and already reported on various conferences and the Pan-American Union), some defenders of nature already brought with them international training and professional experience. Carvalho and Lutzenberger, for example, were trained abroad. Carvalho earned his master’s and doctoral degrees in the United States in the 1940s, while Lutzenberger worked in Germany, Venezuela, and Morocco while employed at BASF, a chemical fertilizer and agrochemical company (Carvalho, 1969; Silva, 2017; Urban, 2011, 2001). The Brazilian environmental scenario was enriched by the exchange of knowledge between scientists and the strengthening of institutions, societies, and associations for natural preservation and conservation, creating an environment that fostered the emergence of the FBCN in 1958.

## The creation of the FBCN and its initial activities

Here we examine the founding of the FBCN. We define its ideals and members, its position, its main objectives, and the process and time involved in its creation and organization. We map out the staff of the FBCN and how they were distributed, and examine the international influences that helped shape the organization, considering its affiliation with the IUCN. Finally, we highlight some of the FBCN’s early activities and how the presence of individuals with social and political influence made these activities possible.

The founders of the FBCN were concerned with the environmental degradation that was taking place in the country and the lack of response by the government, by “more enlightened” citizens, and to a certain extent, society in general. Although it was not the first to be concerned with nature conservation, participants in this group were certain it would be a great success. At this point, some of its members had experience with environmental organizations and policies. Its creation and headquarters in Rio de Janeiro (still Brazil’s capital at that time) was reported in the *Correio da Manhã* from 1958, even though its bylaws were only legally approved in 1960. In 1958 its founders met for the first time, including the singer and journalist Rossini Pinto (1937-1985) and the biologist and journalist Fuad Atala (1933-2019). Pinto regularly wrote a column entitled “Parks and Gardens” in the newspaper, while Atala was a contributor to the section “A bit of science” (“Um pouco de Ciência”) (Atala, 22 jun. 1958, 1 mar. 1959; Franco, Drummond, 2019; Fundação..., 15 jan. 1960; Registro..., 1959; Pinto, 13 jun. 1958, 13 jul. 1958, 31 ago. 1958).

This was not the first time the press published on topics related to the environment in Brazil: Armando Magalhães Corrêa ensured that information from the Sociedade dos Amigos das Árvores was published in the *Correio da Manhã*, and Henrique Luís Roessler regularly published chronicles on nature conservation in the *Correio do Povo*. The members of the FBCN made sure that their ideas and initiatives were featured in the *Correio da Manhã*. According to Nogueira Neto, “our voice was greatly amplified by the newspapers.”^1^ Having journalism as an ally proved effective for disseminating the nature conservation strategies of the FBCN and other institutions (Franco, Drummond, 2009a; Urban, 2001, 2011, p.305).

According to the *Correio da Manhã*, the first meeting of the future foundation was held on May 29, 1958, at the home of the agronomist Harold Edgard Strang, and included the presence of Wanderbilt Duarte de Barros (1916-1997), Francisco Carlos Iglésias de Lima, Victor Abdennur Farah, Eurico de Oliveira Santos (1883-1968), Rosalvo de Magalhães (1930-2005), Rossini Pinto, and Fuad Atala. The *Correio da Manhã* (and later the FBCN, in its first bulletin) reported that there was a variety of associates: “... forestry technicians, naturalists, university academics, excursionists, and journalists, and all interested parties were able to join regardless of political or religious beliefs or race” (Pinto, 13 jun. 1958, p.10) in order to “preserve the fauna, flora, and landscape of our country” (Sociedade..., 1958, p.6). At its core, the FBCN brought together everyone who was concerned with nature and had no political or party connections, despite the political influence wielded by some of its members.

The FBCN’s bylaws were defined quickly. Its members, who were aware of Augusto Ruschi’s interest and active participation in natural conservation, told him of the new planned organization. Ruschi had already structured and founded the SBPN in 1954, and offered to provide the FBCN with all the documentation “spanning preliminary drafts of the bylaws he had written as well as enrollment by characters and entities interested in the initiative” (Atala, 22 jun. 1958, p.1). In this way, the SBPN was extinguished to make way for the FBCN.

Ruschi’s contributions facilitated the process of creating the new organization. Roughly two months after the first meeting to create the foundation, its draft bylaws were ready, and discussed in August 1958 during a new meeting at the home of the agronomist Arthur de Miranda Bastos (1900-1968) for submission to the office of the District Attorney of the Federal District^2^ (Pinto, 13 jun. 1958, 13 jul. 1958). The members of the FBCN approved the bylaws on September 5, 1958, lacking only official filing with the registry office. Private individuals as well as entities affiliated themselves, including Esso, the Brasil-Central Foundation, the senator Jerônimo Coimbra Bueno, and the poet Carlos Drummond de Andrade (1902-1987) (Atala, 10 set. 1958, p.10). According to Franco and Drummond (2009b), by 1989 the FBCN had four thousand members, but most were not militants or did not faithfully attend its assemblies. But the foundation did include some important figures and worked together with other environmental institutions and associations for a wider reach.

On October 31, 1958, the members of the FBCN unanimously chose the dentist and manufacturer Luiz Hermanny Filho (1882-1977) to act as interim president of the FBCN. Along with Hermanny Filho, the interim directorship included Victor Abdennur Farah, Fuad Atala, and Rosalvo de Magalhães as the executive director, secretary, and treasurer, respectively (Atala, 2 nov. 1958). Even though it was not yet formally established, the members of the foundation participated in events and activities on its behalf. Farah, who at that time was leading the CFF, took advantage of this role to act as a bridge between the objectives of the FBCN and his political influence, more specifically his proximity to the president of Brazil at that time, Jânio Quadros (Atala, 1 mar. 1959).

In 1958, the FBCN became affiliated with the IUCN. Harold Edgard Strang articulated this very early connection when he went to Athens to participate in the sixth Assembly of the IUCN, in September 1958. A suggestion was made at this event to create a list of the national parks and reserves around the world. Additionally, the IUCN proposed a program to revise elementary school curricula to include teaching the basic elements of conservationism. Strang was charged with putting the foundations of conservationist principles in writing, acting on the FBCN’s Education and Conservation Committee (Atala, 19 out. 1958; FBCN, 1966; Sugerida..., 9 out. 1958).

In November 1958, in conjunction with the Brazilian Excursionism Union (União Brasileira de Excursionismo, UBE), the Radio-Gymnasts Association (Associação dos Rádio-Ginastas), excursion clubs, and scout groups from the Federal District, the FBCN supported a measure that would release government funding as compensation to the owner of the Garrafão Farm, located in the Serra dos Órgãos National Park (RJ), in order to keep this land under the ownership of the park. This pressure had the desired effect; on December 12, 1958, most of the area in the Garrafão Farm, which had been the victim of predatory exploitation, was definitively placed under the jurisdiction of the park (ICMBio, 2008; Parque..., 27 nov. 1958).

In March 1959, members of the FBCN participated in an edition of the Roquette Pinto radio show entitled “Closer to heaven” (“Mais perto do céu”). It was presented by the UBE to discuss various topics related to the problem of conservationism in Brazil. The program also addressed the relationship between conservationists and excursionists (Natureza..., 1 mar. 1959; Amigos..., 6 mar. 1959). Most excursionists were well-educated members of the middle class who participated in mountain climbing, scouting, spelunking, birdwatching, and orchid cultivation. They were aware of the harmful consequences of developmentalism for nature, which led them to support the foundation. With representatives within the public sphere and connections to civic organizations, the FBCN had the capacity to exert social pressure on governments (Dean, 1996; Lopes, Franco, 2020).

In terms of its activity, the FBCN’s members made it clear that they did not adopt a “romantic” vision of nature “... as an untouchable entity populated with the subjects of visionary lyric poets in love with its beauties. No! Our scope is a realistic policy for wise use of nature, with rational use of its resources” (Conservacionistas..., 1 jul. 1959, p.2). This demonstrates the mixed nature of the FBCN, directed at both stricter preservation of certain areas as well as rational use and conservation of natural resources. In this way, being a nature conservation foundation did not mean it was exclusively conservationist, considering conservationism as a more instrumental perspective for the use of natural resources. It should be noted, the FBCN to a significant degree is located within a tradition influenced by authors such as Henry David Thoreau, John Muir, and Aldo Leopold, one which granted intrinsic value to the natural world, a characteristic of romantic thinking (Franco, Drummond, 2009a; Franco, Schittini, Braz, 2015; Nash, 2001, originally published in 1967; Pádua, 2012; Worster, 2016).

Finally, in June 1959, the founders of the organization filed the *Estatuto da FBCN,* ^3^ the bylaws of the organization, in the registry office. The botanist Fernando Segadas Vianna (1928-2010) was the filing clerk. The 14 founding members were Augusto Ruschi (replaced by Álvaro Silveira Filho when filing the bylaws, as Ruschi was unable to go to the registry office), Luiz Hermanny Filho, Victor Abdennur Farah, Jerônimo Coimbra Bueno, Heitor Grillo (1902-1971), Luis Simões Lopes (1903-1994), Harold Edgard Strang, Fernando Segadas Vianna, Wanderbilt Duarte de Barros, Eurico de Oliveira Santos, Francisco Carlos Iglésias de Lima, David de Azambuja (1917-2007), Rossini Pinto, and Fuad Atala. In January 1960, the registry office approved the bylaws and the foundation was officially granted its legal status (Atala, 1 mar. 1959, 5 jul. 1959; Fundação..., 15 jan. 1960; Registro..., 28 jun. 1959).

The members of the FBCN chose the *curupira*, a figure from “Brazilian indigenous folklore,”^4^ as their symbol (Brasil, 9 abr. 1960). The *curupira* (also *currupira, corupira, caipora, caapora*) can be male or female, with black skin and hair that can turn to fire, with one or two legs, but its feet are always turned backwards in order to confuse anyone who follows its trail. It lives in the forest and protects fauna and flora, knocking its axe against the trees and scaring hunters. The narratives about the *curupira* occasionally mix with other legends, such as *saci-pererê* and the *negrinho do pastoreio* (Anchieta, 1997, originally published in 1560; Cascudo, 2012, originally published in em 1947; Munduruku, 2010).

According to the FBCN, the *curupira* represented what the human view of nature could or should be:

In adopting [the *curupira*] as its symbol, the Brazilian Foundation for Nature Conservation hopes that this myth reemerges among us, not as a pure and simple belief, but because of the proper philosophical spirit it represents, of a life with harmony between man and nature. May it encourage us in every way, especially in terms of work and intelligence, to promote the conservation and renovation of Brazil’s natural resources for future generations (Brasil, 1960).

Also according to the FBCN’s bylaws, the foundation was created without a defined duration, featuring the following main objectives:

(a) Establish parks and national monuments as well as refuges and reserves for native fauna and flora, with special attention to endangered species; (b) Stimulate and promote cooperation between governments and domestic and international organizations interested in conservation of natural resources; (c) Conduct and promote research related to nature conservation; (d) Disseminate conservationist knowledge through courses, competitions, publications, lectures, and conferences; and (e) Plan and carry out activities in the pursuit of this cause (Brasil, 1960).

According to its bylaws, the members of the FBCN were divided into founders, trustees, donors, patrons, sponsors, collaborators, and benefactors. The foundation included a general assembly, presidency, superior council, executive directorship, general secretary, and treasury (Atala, 22 maio 1960; Brasil, 9 abr. 1960; Utilidade..., 12 maio 1960). The election of the defining directorship for its second three-year period, scheduled for March 1959, was only held on April 5, 1960, during the first regular general assembly to elect the president of the superior council. For its president during 1960-1963, the FBCN members unanimously elected Jerônimo Coimbra Bueno (at that time a senator representing the state of Goiás and the União Democrática Nacional party). The superior council was composed of eight members: Luís Simões Lopes, Heitor Grillo, Wanderbilt Duarte de Barros, David de Azambuja, Harold Edgard Strang, Francisco Carlos Iglésias de Lima, Fernando Segadas Vianna, and Eurico de Oliveira Santos. The founders recognized Hermanny Filho as an honorary president because of his prior activities on behalf of the entity (Amigos..., 7 abr. 1960; Assembleia..., 3 abr. 1960; Atala, 27 mar. 1960, 3 abr. 1960).

Coimbra Bueno played a very important role for the FBCN due to his concerns with the environmental cause and his capacity for political influence. These two characteristics of Coimbra Bueno as well as other founding members such as Farah paved the way for the FBCN’s initial plans to create protected areas to be put into practice even before this organization began more intense activities, which only began in 1966. Between its founding and that time, the FBCN built up its membership and activities through smaller, isolated activities mainly thanks to the efforts and social and political influence of some of its members.

## The work of Jerônimo Coimbra Bueno and Victor Abdennur Farah in creating national parks and the 1965 Forest Code

Coimbra Bueno and Farah were the two members responsible for the FBCN’s first successes related to creating protected areas. Because of their proximity to the presidents at that time (Coimbra Bueno with Kubitschek and Farah with Jânio Quadros), 11 national parks were created. Farah also helped draft and edit the 1965 Forest Code (Law 4771 of September 15, 1965), which made it possible to achieve one of the FBCN’s main objectives: establish a national system for nature protection. This section describes the efforts of Coimbra Bueno and Farah and discusses the legacy of their contributions for society and for the FBCN.

In 1959, Coimbra Bueno, who had not yet been elected president of the FBCN, was able to organize the creation of Araguaia National Park (originally established as Ilha do Bananal National Park). It is important to note that part of the reason the park could be created was because conservation was associated with development and the creation of protected areas. Even though Coimbra Bueno intended to leave the park’s area untouched, providing infrastructure only outside of it, then-president Kubitschek associated the process of conservation with profits from activities such as tourism within the park. Despite evidence of an association between conservation, creation of national parks, and economic development, the idea of creating untouched protected areas again implies that in Brazil conservation was not differentiated from preservation. It also evoked an influence of romanticism and the intrinsic value of nature, since natural parks at that time prioritized nature. But almost a decade after its creation, the park contained a luxury hotel and was inhabited by Indians and destroyed by cattle ranches (De Bont, Schleper, Schouwenburg, 2017; Lopes, Franco, 2020; Ribeiro, 2020; Wakild, 2020).

Coimbra Bueno was born in Rio Verde, Goiás, and in 1929 began his studies in civil engineering at the Polytechnic School of Rio de Janeiro, where he graduated in 1933 with a specialty in urbanism. Still at the beginning of his career, he gained political strength when he took the lead in the project to construct the new capital of the state of Goiás, Goiânia. In 1947, he became governor of the state. With a progressive vision, he was concerned with the unchecked exploitation of natural resources in Goiás. Coimbra Bueno proposed legislative reforms directed at protecting fauna and forest reserves, creating a forest police force, and establishing the Goiás Hunting and Fishing Service (Serviço de Caça e Pesca de Goiás). He also defended the creation of several national parks in the state, starting in four locations: Canal de São Simão, Serra de Caldas Novas, Chapada dos Veadeiros, and Ilha do Bananal. His close relationship with Kubitschek facilitated the creation of the last of these three parks in 1959, along with four more: Ubajara (CE), Aparados da Serra (RS), and Emas (GO) (Lopes, Franco, 2020; Ribeiro, 2020).

Coimbra Bueno met Kubitschek when he was still the mayor of Belo Horizonte in 1940. Kubitschek wanted to reurbanize this city, but Coimbra Bueno advised against it and suggested some isolated urban interventions such as Pampulha Square, which helped Kubitschek in his presidential campaign. He also was the main element behind the UDN’s support for the project to construct Brasília. Because the president already knew him and trusted his work, he signed the documents Coimbra Bueno had prepared to create the suggested national parks (Lopes, Franco, 2020).

The recommendation for Coimbra Bueno to serve as the president of the FBCN considered his experience with the “problems of land and conservationism ... Many of the initiatives in the field of nature conservation were born of original projects or ideas from the new president of the FBCN, highlighting the recent creation of Ilha do Bananal National Park” (Atala, 10 abr. 1960, p.7). On April 9, 1960 the official Federal Congress gazette (the *Diário do Congresso Nacional*) published the FBCN’s Declaration of Principles and its bylaws. During a night session of Congress the previous day, Coimbra Bueno said the following:

I believe that, as a result of transferring the Capital to the Central Plateau [Brasília], our immense natural resources are running the risk of irreparable mutilation like what happened on the coast, which is almost entirely eroded, and various species of fauna and flora that are threatened with complete extinction. In this way, the ‘Brazilian Foundation for Nature Conservation’ emerges at a fortunate moment as an echo of all the concerns that have been said, written, and televised in the press, which has spared no efforts to call the attention of the authorities and the general public in order to develop an essential conservationist mentality in the hearts of our people (Senador..., 10 abr. 1960, p.9).

This declaration revealed other motives for the creation of the FBCN. It recognized the absence of nature conservation policies in the country and a certain apathy among the population with regard to environmental destruction, along with an optimistic support of progress and economic growth (Brasil, 9 abr. 1960). FBCN president Coimbra Bueno’s move to Brasília to serve his term as senator left the foundation’s activities on hold.

The FBCN members met again in 1962 at the home of Hermanny Filho to discuss standards to make the FBCN’s work more dynamic (Vai reunir-se..., 17 jun. 1962). Another meeting was held in 1963 at the headquarters of the National Society for Agriculture, a public institution created in 1897 to promote the development of agriculture in Brazil. At this meeting, new members were approved and a general assembly was convened to select the new directorship (Será revista..., 31 mar. 1963). Coimbra Bueno was reappointed to the presidency of the FBCN, although he regretted that his participation would be minimal due to his congressional activities. However, he emphasized that “during his legislative work he was able not only to obtain resources for the institution but also carry out activities in the areas of laws to defend natural heritage, citing numerous achievements,” probably referring to the fact that he had convinced Kubitschek to create the national parks (Reconduzido..., 6 abr. 1963, p.10).

Another conservationist who used his political and social influence for the FBCN to be effective was the agronomist Victor Abdennur Farah, executive director of the FBCN from 1958 to 1963 and president of the CFF from 1956 to 1967. The CFF played an important role during the administration of president Jânio Quadros (1917-1992), who was sensitive to environmental issues and followed Farah’s suggestions (Lopes, Franco, 2020; Silva, 2017; Urban, 2011). In an interview, Nogueira Neto stated that

Farah was directly received by Jânio Quadros. When he went in, the President rose to his feet and said: ‘Mr. President, please!’ Because he [Farah] was also president of the council. And indeed, the vast majority of the reserve areas that existed up to 1961 in Brazil were created by Jânio Quadros. The Federal Forest Council and Farah had a decisive influence (Urban, 2011, p.224).

Quadros approved the establishment of six national parks during his short tenure: Caparaó (MG), Brasília (DF), Monte Pascoal (BA), São Joaquim (SC), Sete Cidades (PI), and Tijuca (RJ). Farah also was part of the plan to draft the 1965 Forest Code, along with the agronomist Alceo Magnanini (1925-2022) and legal scholar Osny Duarte Pereira (1912-2000). Pereira studied the history of Brazilian forest law, environmental legislation, and the 1934 Forest Code, and introduced the principle that collective interest limits exploitation of private ownership to Brazilian forest law, based on the concept of ownership defined in the 1946 Constitution. This was reflected in the 1965 Forest Code (Franco, 2021; Gonçalves, 2021; Lopes, Franco, 2020; Urban, 2011).

Another factor besides Farah’s efforts that contributed to the development of the 1965 Forest Code was international institutional exchanges by government staff who acquired scientific knowledge and expanding ecological leanings. The 1965 Forest Code defined the foundations for a national system for protecting nature which was only firmly implemented in 2000 with the National System of Conservation Units (SNUC) through law 9985 of July 18. In this way, the FBCN’s efforts helped to create the SNUC over the long term.

The work of Coimbra Bueno and Farah was typical of the FBCN, in its early moments as well as over time: cooperation with the government rather than confrontation. Together with institutions and legislation, these conservationists show how the scenario that led to the creation of the FBCN was shaped and strengthened over the preceding decades. Beyond political influence, the members of the FBCN also used technical and scientific arguments to convince the military government to create protected areas. This allowed the foundation to be successful in its nature conservation policies even during the dictatorship, from 1964 to 1985. The FBCN was more active from 1966 onward with the creation of committees (following the example of the IUCN) and as it started publishing its informative annual reports, which intensified the dissemination of data on nature conservation and facilitated convergence and encounters between people interested in this subject.

## Final considerations

The FBCN was not the first environmental NGO created in Brazil, but it had the greatest reach, both domestically as well as in its contacts with foreign conservationist NGOs, institutions, and intellectuals. Although it was established in 1958, it peaked between 1966 and 1989, when it published annual reports and engaged in many domestic environmental conservation projects. Even before this, however, the FBCN was engaged in isolated nature conservation activities.

One reason for the organization’s successes was the influence its founders and members wielded, often within the national government, using their prestige in the political sphere or even the media. The journalists Rossini Pinto and Fuad Atala were largely responsible for publicizing the FBCN’s activities in their columns in the *Correio da Manhã* newspaper. Two other clear examples of political prestige and influence were Jerônimo Coimbra Bueno and Victor Abdennur Farah. Both held public office and were trusted advisors to two presidents, leading to success in their projects to create national and international parks during a period of developmentalist discourse and activities at home and abroad, considering that this was a period of major productive acceleration.

In this way, although the FBCN was not carrying out intense activities during its initial phase (1958-1966), its members were able to exert significant influence within significant government spheres, which remained common among many of its members throughout its existence. Together with the development of scientific knowledge and legislative proposals (in this specific case, the 1965 Forest Code), this strategy of personal and political influence led to the attainment of one of the foundation’s main objectives in the long term and through other environmental tools and policies: the creation of a system of protected areas, which took shape in 2000 via the Conservation Units Law, which is still in effect and a constant topic of discussions on biodiversity conservation and sustainable development.

Paulo Nogueira Neto, a member of the FBCN, was part of the World Commission on Environment and Development, which in 1987 drafted the Brundtland Report. This was the first document to propose the concept of sustainable development and discussed the relationship between humans and the environment, including the question of biodiversity loss. The attention given to preserving threatened species was another subject the FBCN focused on, later culminating in concerns with biodiversity conservation, which involves understanding and establishing activities to avoid extinction and loss of biological diversity in terms of species, genes, and ecosystems. It is important to note that other topics of interest for the FBCN were environmental pollution, population growth, and urbanization.

These environmental concerns were reinforced with the United Nations Conference on Environment and Development, also known as Eco-92 or the Earth Summit, held in Rio de Janeiro in 1992. The FBCN participated in the conference, although by this time it was financially fragile and shared this scene with other domestic and international environmental NGOs. In summary, the FBCN demonstrated from its very beginnings concerns with both the concept of preservation (strictly speaking) as well as the broader concept of conservation, which developed within the sphere of environmental debates over time. Here we note that many FBCN members affirmed the intrinsic value of nature, simultaneously linking them with an older, romantic tradition as well as the science of conservation biology that took shape during the 1980s and the deep ecology proposed by Arne Næss. This aspect was not as evident in the activities of Coimbra Bueno and Farah, who worked politically in the defense of nature. When the ecologist José Cândido de Melo Carvalho became president of the FBCN in 1966, the organization’s activities related to this romantic tradition and conservation biology became clearer as its work increasingly focused on protected areas and rare or endangered species.

## Data Availability

They are not in a data repository.

## NOTES

1 In this and other citations of texts from Portuguese, a free translation has been provided.

2 The Distrito Federal at that time was what is currently the city of Rio de Janeiro.

3 The FBCN was considered an organization of public utility by Law 601 of October 30, 1964, according to the *Diário Oficial da Assembleia Legislativa do Estado da Guanabara* of November 10, 1964. It was registered in Liber A n.5 of the Civil Registry of Corporate Entities in the Sixth Registry Office of the State of Guanabara (FBCN, 1966).

4 The FBCN used the term “folklore,” often used even today to refer to myths and legends; we opted to maintain this term, but it is important to emphasize that there are different understandings of what myth, mythology, legends, and even stories entail. Sales (2014) notes that myths and mythologies are related to creation (of the earth, of humans, of fire etc.) or rites of passage, while legends refer to enchanted beings who are generally connected to nature, with narratives that may vary depending on where they occur. Stories are also related to these enchanted beings, but may surpass geographic and even linguistic barriers. Folklore tends to be mixed with what can be defined as myth or legend.
